# Prefrontal Cortex Activity Predicts Mental Fatigue in Young and Elderly Men During a 2 h “Go/NoGo” Task

**DOI:** 10.3389/fnins.2018.00620

**Published:** 2018-08-31

**Authors:** Asta Terentjeviene, Edita Maciuleviciene, Kazys Vadopalas, Dalia Mickeviciene, Diana Karanauskiene, Dovile Valanciene, Rima Solianik, Arunas Emeljanovas, Sigitas Kamandulis, Albertas Skurvydas

**Affiliations:** ^1^Institute of Sport Science and Innovations, Lithuanian Sports University, Kaunas, Lithuania; ^2^Department of Health, Physical and Social Education, Lithuanian Sports University, Kaunas, Lithuania; ^3^Department of Applied Biology and Rehabilitation, Lithuanian Sports University, Kaunas, Lithuania

**Keywords:** aging, motor fatigue, mental fatigue, prefrontal cortex, executive function

## Abstract

**Background:** Although the effects of mental fatigue on cognitive–motor function and psychological state in young adults are well-documented, its effects in the elderly are not completely understood. The aim of this study was to estimate the effect of prolonged cognitive load on the indicators of psychological, cognitive, and motor functions.

**Methods:** Fifteen young and 15 elderly men were asked to perform a 2 h “Go/NoGo” task. Psychological state (mood and motivation), cognitive (prefrontal cortex activity and cognitive performance), and motor (motor cortex excitability and grip strength) functions were measured before and after the task. During the 2 h task, both groups had a significantly similar increase in the number of “Incorrect NoGo” errors. Only in young men reaction time (RT) of “Incorrect NoGo” and intraindividual variability of RT of “Incorrect NoGo” significantly increased during task. After the task, handgrip strength decreased for the young men, whereas latency of motor evoked potentials prolonged both groups. Nevertheless, both groups indicated that they felt fatigue after the 2 h task; we observed that mental demand increased, whereas intrinsic motivation and mood decreased only in young men. Prolonged task decreased the switching/rest ratio of oxygenated hemoglobin for the young and the elderly men; however, greater for elderly than young men. Interestingly, the more the prefrontal cortex was activated before the 2 h task during the switching task, the fewer of “Incorrect NoGo” errors made by the young men and the greater the number of errors made by the elderly men.

**Conclusion:** Because of the greater mental load and (possibly) greater activation of prefrontal cortex during the 2 h “Go/NoGo” task, there was greater mental and neuromuscular performance fatigue in young men than in elderly men.

## Introduction

Functional limitations, disabilities, mortality, and other adversative consequences in the elderly can be highly predicted by fatigue (Skurvydas et al., 2011; Ishii et al., 2014). Mental fatigue is first of all the subjective feeling of a worsened ability to engage in mental activities, but it can also be measured objectively by decreased performance (Lorist, 2008; Solianik et al., 2016; Skurvydas et al., 2017; Chuang et al., 2018). There is a lack of understanding of the cognitive mechanisms of mental fatigue origin. It is still not clear whether the decreased performance due to mental fatigue is caused by a continuous deterioration of the cognitive properties (e.g., attention and memory) (Baumeister, 2014) or by a scarce enrolment of intact cognitive processes, caused by the decrease in motivation (Chaudhuri and Behan, 2000). Botvinick and Braver (2014) suggest that mental fatigue has a control mechanism that expels people from lengthy tasks and pushes them to newer and supposedly more rewarding activities. They established that after mental fatigue, increasing extrinsic motivation recovered the level of performance which was before the fatigue, and maintained that this provided evidence in support of a fatigue-induced detachment from the task (Botvinick and Braver, 2014). Chaudhuri and Behan (2000) suggest that mental fatigue might be caused by decreased motivation to take part in self-initiated activities and it is a consequence of changes in the motivational brain circuits, together with the basal ganglia.

The self-control mechanism is highly dependent on the so-called executive function, which operates effectively if concentration on the required object and inhibition of unnecessary objects (temptations), working memory, and flexible switching of attention work well (Diamond, 2013). The main controller of the executive function (and self-control) is localized in the prefrontal cortex of the brain, which is responsible for the management of cognitive tasks and emotions (Buschman and Miller, 2014). One of the most important characteristics of self-control is the ability to inhibit undesirable stimuli, such as temptations (Baumeister, 2014). Cognitive fatigue was caused by continued performance of tasks which were cognitively demanding (compared with controls) (Klaassen et al., 2014). Results showed that age affected the left dorsolateral prefrontal and superior parietal cortex activation during working memory encoding; also greater activation was more pronounced among middle-aged than young adults irrespective of the load of the working memory or the condition of fatigue (Klaassen et al., 2014).

“Go/NoGo” and “Stroop” test exercises are widely used for inducing cognitive fatigue (Netz et al., 2016; Solianik et al., 2018). These and similar exercises usually last from 30 min to several hours and cause a decrease in mental working capacity (Shigihara et al., 2013). It has been proven that the “Go/NoGo” task requires self-control skills, the main goal of which is to inhibit redundant tasks (Brown et al., 2015; Netz et al., 2016).

The speed of processing, working memory, inhibitory function, and long-term memory decline with age, similarly, the brain structure size and white matter integrity decrease (Park and Reuter-Lorenz, 2009). The age-related compensatory recruitment of prefrontal cortex during cognitive (Emery et al., 2008; Park and Reuter-Lorenz, 2009) and motor controls (Seidler et al., 2010) tasks has been established. Older adults demonstrate greater activation when they perform tasks that engage executive functions, episodic memory, and working memory tasks, compared with young adults (Emery et al., 2008). Older adults show more widespread involvement of brain regions responsible for motor control than young adults, principally the prefrontal cortex and basal ganglia networks (Seidler et al., 2010). These regions are the most susceptible to age-related effects, which results in a disparity between “supply and demand.” However, other elements of these compensatory mechanisms and their findings reflecting cognitive decline must be thoroughly investigated in the future.

The main aim of our studies was to test the following hypothesis. As older adults more highly activate executive, cognitive, and association brain regions aiming at performing the tasks (Emery et al., 2008; Park and Reuter-Lorenz, 2009; Seidler et al., 2010; Klaassen et al., 2014), (1) elderly men should have greater fatigue in a “Go/NoGo” task lasting 2 h than younger men; (2) they should make more errors, there should be a greater increase in the variability of task performance, a greater decrease in neuromuscular function, a greater increase in the subjective feeling of fatigue and greater effort during exercise, a greater decrease in cognitive function, while motivation during the task should not be different between the young and elderly men; and (3) objective and subjective indicators of fatigue both in young and elderly men should depend on prefrontal cortex activity as measured by functional near-infrared spectroscopy (fNIRS).

## Materials and Methods

### Subjects

Thirty healthy participants took part in this study: 15 young [age: 22.2 ± 2.7 years, height: 180.1 ± 6.3 cm, body mass index (BMI): 23.5 ± 2.5 kg/m^2^] and 15 elderly (age: 72.7 ± 5.7 years, height: 176.3 ± 4.8 cm, BMI: 25.1 ± 3.1 kg/m^2^) men. Young and elderly participants were healthy, non-smokers, and right-handed (confirmed using the Edinburgh Handedness Inventory). Participants were asked not to consume caffeine and alcohol-containing products 12 h before the experiment, and were told to come another time if they were ill or did not sleep well the night before the experiment. Written informed consent was obtained from all participants after explaining to them all the details of the experimental procedures as well as potential discomforts and risks. The studies were approved by the local Ethics Committee (The Kaunas Regional Ethics Committee, No. BE-2-40), performed in accordance with the Declaration of Helsinki.

### Rationale of Experiment

**FIGURE 1:**
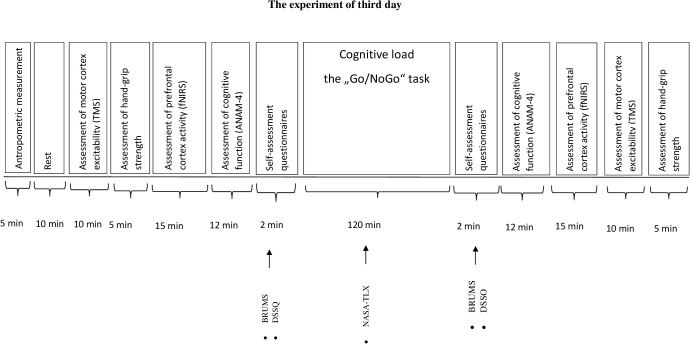
The experiment of third day. BRUMS, Brunell Mood Scale; DSSQ, Dundee Stress State Questionmaire; NASA-TLX, National Aeronautics and Task Load Index.

Participants came to the laboratory three times. During the first and the second visit (day), participants were familiarized with the experimental procedures (cognitive and motor performance tests, assessment of motor cortex excitability during TMS). Besides, on the second day the subjects filled in a self-assessment questionnaire: the Schutte Self-Report Emotional Intelligence test (SSREIT). The third day protocol is given in **Figure 1**. Each participant accomplished all three visits during 2 weeks with a minimum of 48 h recovery period between each visit. Before the start of the experiment (the third day), the participants were asked to sleep for 8 h the night before the experiment, and to avoid ingesting alcoholic beverages, caffeine, and sedating antihistamines for 48 h and from heavy exercise for at least 24 h before the experiment. The experiment of the third day was performed at 8:00 in the morning.

### Measurements

#### Assessment of Prefrontal Cortex Activity

The fundamentals of fNIRS are described in detail in other sources (Ferrari and Quaresima, 2012). Assessment of brain activity was performed on a continuous wave system (fNIR Imager 1100, fNIR Devices LLC, Potomac, MA, United States) using a flexible 16 optode probe set. The sensor has a temporal resolution of 500 ms per scan with a 2.5 cm source-detector separation allowing for approximately 1.25 cm penetration depth and 16 measurement locations on a rectangular grid covering the forehead region, designed to observe dorsal and inferior frontal cortical areas (Ayaz et al., 2013). Two different wavelengths (730 and 850 nm) are used by the system, and its frequency is controlled for wavelengths and channels to avoid cross talk. COBI Studio software was used for data acquisition (Ayaz et al., 2013). The signals of all channels were verified before recording. Data analysis was performed using fNIRSoft analysis software (BIOPAC Systems, Inc., United States). Oxygenated hemoglobin (OxyHb) values were calculated from raw data by solving a modified Beer–Lambert equation. Data were filtered to remove physiological and other artifacts. The changes of OxyHb were acquired from all the participants in all 16 channels and the data were averaged. Prefrontal cortex activity was assessed during the 5 min rest and during the Switching Task.

#### Assessment of Cognitive Function

Cognitive function was assessed using a computerized Automated Neuropsychological Assessment Metric, version 4 (*ANAM-4;* Center for the Study of Human Operator Performance University of Oklahoma, Norman, OK, United States), a reliable screening instrument designed for repeated evaluations (Reeves et al., 2007). The test battery took about 12 min to perform. The chosen tests measured the task accuracy (percent of correct responses) and mean response time (mean reaction time, RT).

##### The Switching Task (ST)

This task measures the ability of mental flexibility and shifting set (Reeves et al., 2007). It is a combination of the Mathematical Processing Task (MPT) and the Manikin Task (MT). The MT is located on the left side of the computer screen and the MPT is located on the right side of the computer screen, and the user is guided by means of a red arrow at the bottom of the screen to respond to the problem on the left or the right side. Responses are inserted by using the keyboard, when the left hand is used for the MT and the right hand is used for the MPT. This test consisted of 64 trials.

##### The Simple Reaction Time Task (SRTT)

This test measures Simple Reaction Time (SRT) by providing the participant with a series of “^∗^” symbols on the display. The participant is instructed to respond as quickly as possible by pressing a button as soon as the stimulus appears. This test consisted of 40 trials. Results of this test are used as a measure of attention, visuo-motor response planning, and timing.

##### The Code Substitution and Code Substitution Delayed Tasks (CSIT and CSDT)

This test measures attention, concentration, and learning. During this test, nine symbols and nine numbers are paired with a unique number located below a specific symbol. The participant is instructed to try to remember the symbol–number pairs because they will be asked to recall them later. During the learning phase, the participant indicates whether or not the pairings at the bottom match the key and receives feedback for incorrect responses, if the pair is correct, the participant presses the left mouse button; if incorrect, the right mouse button. During the recall phase, there is no key at the top and the participant must indicate if the pairings appearing at the bottom are correct or incorrect from memory. This test consisted of 40 trials.

##### The Two-Choice Reaction Time Task (TCRTT)

This task measures ability to shift mental set (mental flexibility). During this test, one of two stimuli is presented on the screen (“^∗^” or “o”) with a variable interstimulus. The participant is instructed to respond as quickly as possible by pressing the left mouse button each time the “^∗^” stimulus is presented or the right mouse button each time the “o” stimulus is presented. This test consisted of 40 trials. Results of this test are used as a measure of processing speed and alternating attention with a motor speed component.

##### The Mathematical Processing Task (MPT)

This task measures working memory. During this test, an arithmetic problem requiring an addition and subtraction of three single-digit numbers is displayed (e.g., “5 – 2 +3 = ”). The participant is instructed to respond as quickly as possible by pressing the left mouse button if the answer to the equation is greater than 5 or the right mouse button if the answer is less than 5. The correct answer may be any number from 1 to 9 except 5. This test consisted of 20 trials. Results of this test are used as an index of basic computational skills, concentration, and working memory.

##### The Matching Grids Task (MGT)

This task measures visuospatial discrimination. During this test, two 4 × 4 grids are displayed side by side on the screen; however, one 4 × 4 pattern is rotated. The participant is instructed to indicate as quickly as possible if the grids are exactly the same, except for a possible rotation, and to click the left mouse button and the right mouse button if the grids are different. This test consisted of 20 trials. Results of this test are used as an index of visuospatial processing.

##### The Pursuit Tracking Task (PTT)

This task measures visuomotor control. During this test, the participant is instructed to move the computer mouse so that the cursor tracks a moving target with a “+” symbol inside. The mouse cursor is required to remain inside the box and be kept as close to the symbol as possible as it moves across the screen in a circular pattern for 2 min. The path and the accuracy of movement are established.

##### The Manikin Task (MT)

This task measures spatial orientation ability. During this test, a figure of a man is presented holding a ball in one hand and a cube in the other hand, and a ball or a cube is displayed at the bottom of the screen. The figure of the man appears in various orientations: standing upright or upside down and either facing toward the test taker or away. The participant is instructed to indicate as quickly as possible which of the man’s hands is holding the object displayed at the bottom on the screen and to press the left mouse button if the answer is left and the right mouse button if right. This test consisted of 32 trials. This test assesses three-dimensional spatial rotation ability, left–right orientation, problem solving, and attention.

##### The Memory Search Task (MST)

This task is an adaptation of Sternberg’s memory search/serial reaction time task, which measures verbal working memory. During this test, a string of six letters is presented for memorization. The participant is instructed to press the space bar once the string has been memorized; then, it disappears from view and individual letters are presented one at a time. The participant is instructed to indicate as quickly as possible whether the letter belongs to the memorized set and press the left mouse button for letters included in the memory set and the right mouse button for those not. This test consisted of 40 trials. Results of this test are used as an indicator of verbal working memory, immediate recognition, and attention.

#### Cognitive Load – The “Go/NoGo” Task

The *“Go/NoGo” task* measures response inhibition (Chikazoe, 2010). During this test, a participant is required to respond to a go stimulus as quickly as possible, but is required to withhold a response to a no-go stimulus. During this test, 5400 stimuli appeared on the computer screen for each research participant: 4320 of them were “Go” stimuli and 1080 – “NoGo” stimuli (“Go” stimuli occurred in 80% of trials, with “NoGo” stimuli occurring in 20%.). As soon as the participant reacted to the stimulus on the screen, a new stimulus appeared immediately. The duration of the test was 120 min, 8 series, 15 min each (2–3 s between series). We established the following indicators: “Correct Go” RT, “Incorrect NoGo” RT, “Correct Go after Correct NoGo” RT, number of correct and incorrect responses (“Incorrect Go”), intraindividual variability [coefficient of variation (CV)] of “Correct Go,” “Incorrect NoGo,” “Correct Go after Correct NoGo.” During 2 h “Go/NoGo” task the participant filled in the National Aeronautics and Space Administration Task Load Index (NASA-TLX) questionnaire every 30 min. It took about 2 min.

#### Assessment of Motor Cortex Excitability During Transcranial Magnetic Stimulation (TMS)

EMG was recorded from the abductor pollicis brevis (APB) muscle with a motor evoked potentials (MEP) monitor (MagVenture A/S, Denmark) with 26 mm diameter pregelled disposable Ag/AgCl electrodes (FIAB, Italy) placed on clean skin. EMG recordings were band-pass filtered (20–5000 Hz), sampled at 100 ks/s, digitized, and stored in a computer for analysis. A Magpro X100 transcranial magnetic stimulator (MagVenture A/S, Denmark) with a handheld figure-of-eight coil (95 mm D-B80 Butterfly Coil; MagVenture A/S) was used to elicit MEPs in the right ABP muscle. Single pulses (biphasic waveform, pulse width 280 μs) were delivered manually. The optimal coil position was determined before the experiment as follows. Using a slightly suprathreshold stimulus intensity, the coil was moved to determine the optimal point on the left primary motor cortex for stimulation, from which maximal amplitude MEPs were elicited in the ABP muscle. The coil was placed tangentially to the scalp with the handle pointing backward and laterally at a 45° angle away from the midline. Then the position of the coil was marked on a latex swimming cap for correct repositioning. The stimulator output intensity was adjusted to 130% of resting motor threshold (rMT), which was defined as the minimal intensity of stimulator output that produces MEPs with amplitudes of at least 50 mV with 50% probability (Rossini et al., 1994). The subjects were constantly monitored to ensure absence of voluntary APB muscle contraction. A total of 10 stimuli were collected at approximately 10 s intervals, and averaged for analysis. The most important indicators were latency and amplitude of MEP.

#### Assessment of Hand-Grip Strength

In the study, a hydraulic hand dynamometer (Jamar, Lafayette Instrument Company, United States) was used to measure isometric hand-grip strength. After the maximal hand grip, maximal hand-grip strength (0–90 kg) was shown on the screen. For each subject, the device handle was adapted according to the size of their hand. With the dominant hand (all subjects were right handed) the subject performed the testing with Jamar hydraulic hand dynamometer twice: before “Go/NoGo” task and after the 2 h “Go/NoGo” task. The subject was seated in a chair with their arm pointing to the front, the elbow bent at 90°. The subject was allowed three trials, the best result was recorded.

#### Self-Assessment Questionnaires

##### The Schutte Self-Report Emotional Intelligence Test (SSREIT)

To measure the emotional intelligence of participants, the SSREIT (Schutte et al., 1998) was used. The SSREIT is a self-report inventory consisting of 33 items scored on a five-point Likert scale (1 = strongly disagree, 2 = disagree, 3 = neither disagree nor agree, 4 = agree, 5 = strongly agree). All these items can be divided into four subscales: perception of emotion (e.g., “When I am faced with obstacles, I remember times I faced similar obstacles and overcame them”), managing own emotions (e.g., “I am aware of the non-verbal messages I send to others”), managing others’ emotions (e.g., “I know when to speak about my personal problems to others”), and utilization of emotions (e.g., “When my mood changes, I see new possibilities”). Total scores can range from 33 to 165, where a higher score indicates a higher quality of emotional intelligence.

##### The Brunel Mood Scale (BRUMS)

Current mood (“How do you feel right now?”) was assessed with the Brunel Mood Scale (BRUMS) (Terry et al., 2003). This questionnaire contains 24 items (e.g., “angry,” “uncertain,” “miserable,” “tired,” “nervous,” and “energetic”) divided into six respective subscales: anger, confusion, depression, fatigue, tension, and vigor. The items were answered on a five-point Likert scale (0 = not at all, 1 = a little, 2 = moderately, 3 = quite a bit, 4 = extremely), and each of subscales with four items can summed for a total score of 0 to 16. Higher scores on each subscale represent a greater current mood extreme (anger, confusion, depression, fatigue, tension, or vigor) of the participants.

##### The Dundee Stress State Questionnaire (DSSQ)

The Dundee Stress State Questionnaire (DSSQ) thinking content and motivation scales were used (Matthews and Desmond, 2002). The thinking content scale related to task performance was measured on subscales of the DSSQ: task-related interference (eight items) and task-irrelevant interference (eight items). The scale consists of 16 items (e.g., “I thought about how I should work more carefully” and “I expect the content of the task will be interesting”) scored on a five-point Likert scale (0 = not at all, 1 = a little bit, 2 = somewhat, 3 = very much, 4 = extremely). Therefore, total scores for each thinking content subscale range between 0 and 32, where a higher score indicates higher thinking content. The DSSQ motivation scale was used to assess motivation related to “Go/NoGo” task performance. The motivation scale comprises 15 items that include groups of three-dimensional questions: questions about success motivation (want to perform actions good or better than others), questions about intrinsic motivation (to be interested), and one question about overall motivation. The scale consists of two subscales: success motivation (seven questions) and intrinsic motivation (seven questions) (e.g., “I wanted to succeed on the task” and “I felt apathetic about my performance”). The scale is scored on a five-point Likert scale (0 = not at all, 1 = a little bit, 2 = somewhat, 3 = very much, 4 = extremely). Therefore, total scores for each motivation scale range between 0 and 28, where a higher score indicates higher motivation.

##### The National Aeronautics and Space Administration Task Load Index (NASA-TLX)

To evaluate the subjects’ perceived workload and performance during 2 h “Go/NoGo” task, participants were asked to respond to the NASA-TLX questionnaire, which includes six dimensions: mental demand (“How much mental and perceptual activity was required?”), physical demand (“How much physical activity was required?”), temporal demand (“How much time pressure did you feel due to the rate or pace at which the tasks or task elements occurred?”) and perceived performance (“How successful do you think you were in accomplishing the goals of the task set by the experimenter?”), effort (“How hard did you have to work to accomplish your level of performance?”) and frustration (“How insecure, discouraged, irritated, stressed and annoyed versus secure, gratified, content, relaxed and complacent did you feel during the task?”) (Hart and Staveland, 1988). The participants scored each of the items on a scale divided into 20 equal intervals anchored by a bipolar descriptor (e.g., high/low). This score was multiplied by 5, resulting in a final score between 0 and 100 for each of the subscales, where a higher score indicates higher overall workload.

### Statistical Analysis

The data were tested for normal distribution using the Kolmogorov–Smirnov test, and all data were found to be normally distributed. A two-way mixed analysis of variance (ANOVA) with age as a between-group factor and with time as a within-group factor was taken. If significant effects were found, Sidak’s *post hoc* adjustment was used for multiple comparisons across a set of conditions within each repeated-measures ANOVA. Statistical significance was defined as *p <* 0.05. Together with this, calculations for statistical power [observed power (*OP*)] were performed and the partial eta squared ($\eta_{\text{p}}^{2}$) was estimated as a measure of the experimental trial effect size. Pearson correlation coefficients (*r*) were used to identify relationships between variables. Statistical analyses were performed using IBM SPSS Statistics software (v. 22; IBM Corporation, Armonk, NY, United States).

## Results

### “Go/NoGo” Task

**FIGURE 2:**
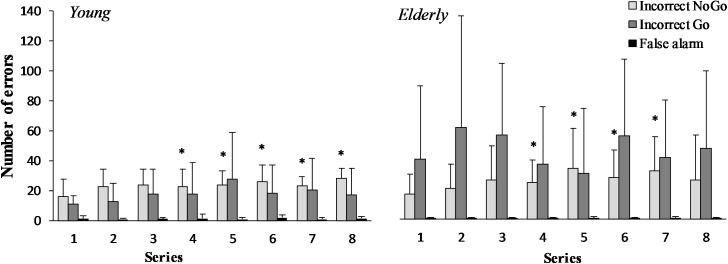
“Incorrect NoGo,” “Incorrect Go,” “False alarm” errors during 2 h “Go/NoGo” task in young and elderly men. *^∗^p* < 0.05 compared with the first series.

There was a significant increase in the number of “Incorrect NoGo” errors in the young men and the elderly men during the “Go/NoGo” task (*F*_(7,196)_ = 3.43; *p* = 0.003; *OP* = 0.95; $\eta_{\text{p}}^{2}$ = 0.22) (**Figure 2**). However the effect of age was not significant (*F*_(1,29)_ = 0.26; *p* = 0.619; *OP* = 0.076; $\eta_{\text{p}}^{2}$ = 0.021). The “Incorrect Go” error depends neither on task performance (*F*_(7,196)_ = 0.66; *p* = 0.708; *OP* = 0.27; $\eta_{\text{p}}^{2}$ = 0.05), nor age (*F*_(1,29)_ = 3.34; *p* = 0.094; *OP* = 0.38; $\eta_{\text{p}}^{2}$ = 0.21). “False alarm” error did not change significantly during 2 h “Go/NoGo” task (*F*_(7,196)_ = 0.64; *p* = 0.719; *OP* = 0.26; $\eta_{\text{p}}^{2}$ = 0.05) and there were no significant differences between young and elderly men (*p* > 0.05) in all cases interaction effect of task and age was not significant (*p* > 0.05).

**FIGURE 3:**
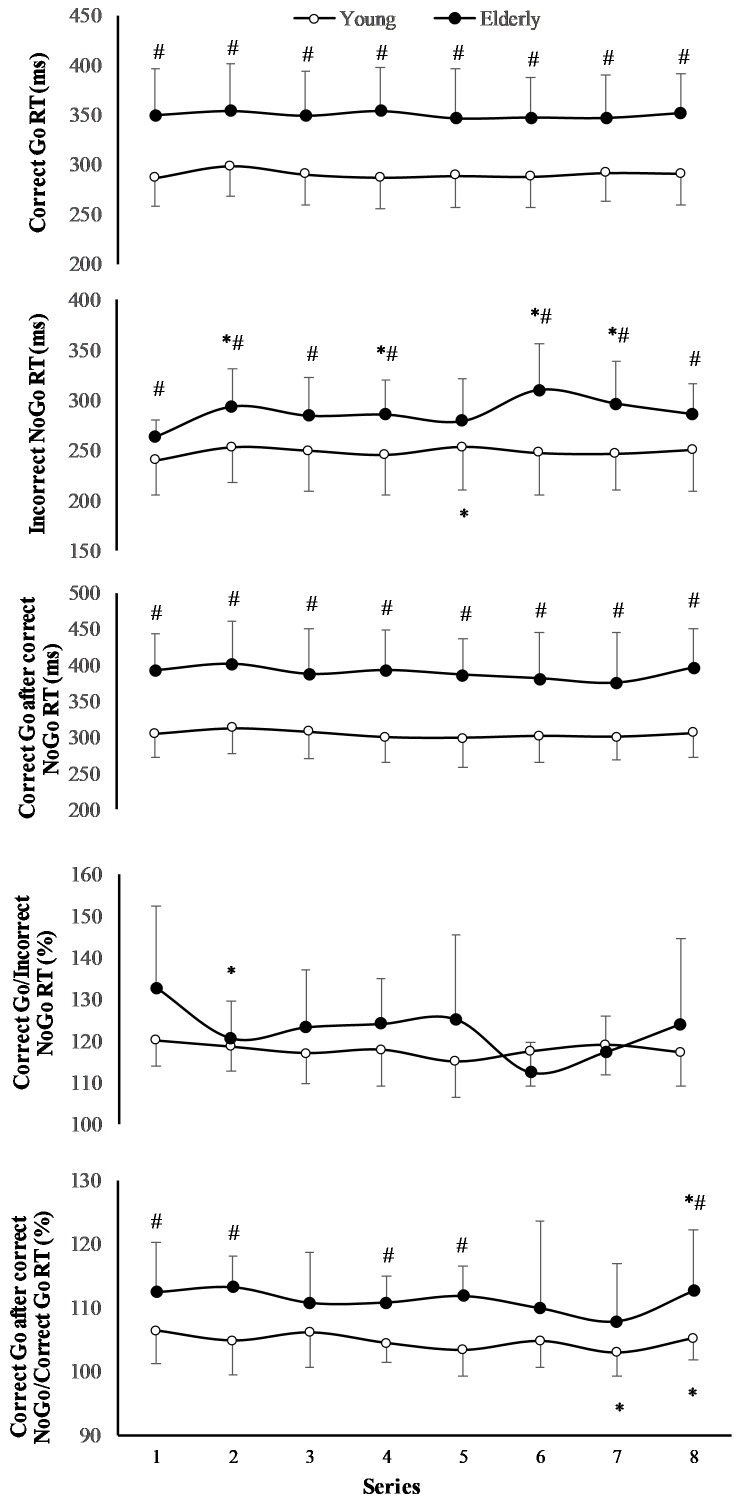
Reaction time (RT) during 2 h “Go/NoGo” task. *^∗^p <* 0.05 compared with the first series; *#p* < 0.05 compared with the young men.

The RT of “Correct Go” did not significantly depend on the task (*F*_(7,196)_ = 0.71; *p* = 0.667; *OP* = 0.29; $\eta_{\text{p}}^{2}$ = 0.06), but it significantly depended on age (*F*_(1,29)_ = 6.77; *p* = 0.024; *OP* = 0.69; $\eta_{\text{p}}^{2}$ = 0.36) (**Figure 3**). The RT of “Incorrect NoGo” significantly depended on the task (*F*_(7,196)_ = 2.47; *p* = 0.02; *OP* = 0.85; $\eta_{\text{p}}^{2}$ = 0.18) and on age (*F*_(1,29)_ = 2.98; *p* = 0.012; *OP* = 0.55; $\eta_{\text{p}}^{2}$ = 0.19). Only in young men RT of “Incorrect NoGo” significantly increased during 2 h “Go/NoGo” task (*p* < 0.05). The after “Correct NoGo” RT of “Correct Go” did not significantly depend on the task (*F*_(7,196)_ = 1.57; *p* = 0.154; *OP* = 0.52; $\eta_{\text{p}}^{2}$ = 0.11), but it depended on age (*F*_(1,29)_ = 9.78; *p* = 0.009; *OP* = 0.82; $\eta_{\text{p}}^{2}$ = 0.45). Moreover, the ratio of RT of “Correct Go” to “Incorrect NoGo” depended significantly neither on the task nor on age (*p* > 0.05). We found that the ratio of RT of “Correct Go” “after correct NoGo” to “Correct Go” did not significantly depend on the task (*F*_(7,196)_ = 6.71; *p* = 0.137; *OP* = 0.49; $\eta_{\text{p}}^{2}$ = 0.12), but it depended on age (*F*_(1,29)_ = 4.97; *p* = 0.041; *OP* = 0.69; $\eta_{\text{p}}^{2}$ = 0.29) In all cases the interaction effect of age and task was not significant (*p* > 0.05).

**FIGURE 4:**
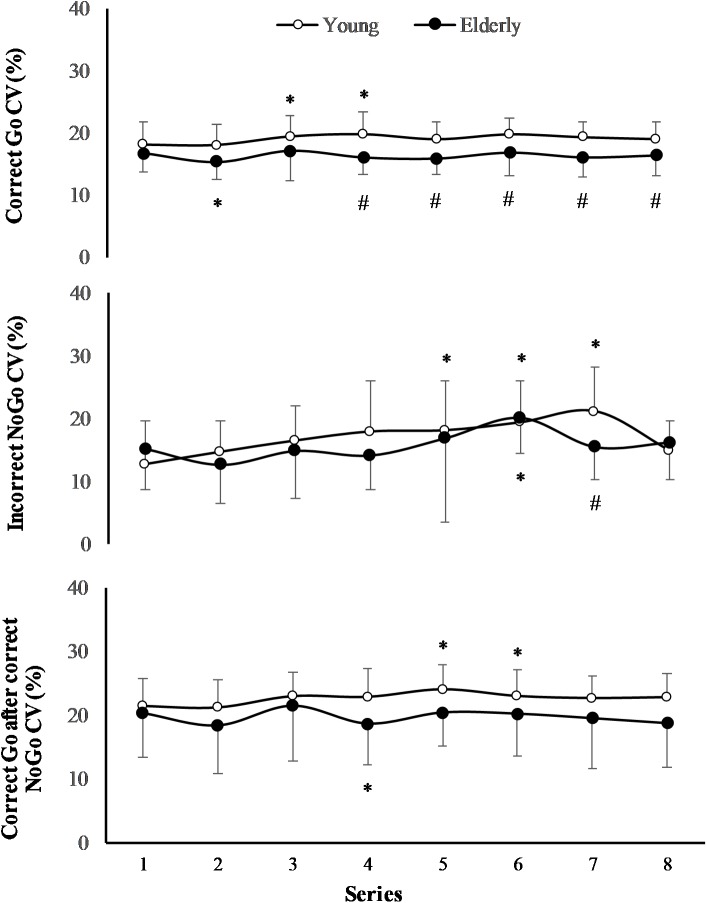
Coefficient of variation (CV) of RT during 2 h “Go/NoGo” task. *^∗^p* < 0.05 compared with the first series; *#p <* 0.05 compared with the young men.

The effect of task on CV of RT of “Incorrect NoGo” was significant (*F*_(7,196)_ = 3.13; *p* = 0.005; *OP* = 0.93*;*$\eta_{\text{p}}^{2}$ = 0.2), however, the effect of age was not significant (*F*_(1,29)_ = 0.78; *p* = 0.39; *OP* = 0.12; $\eta_{\text{p}}^{2}$ = 0.06) (**Figure 4**). Only in young men CV of RT of “Incorrect NoGo” significantly increased during PCL (*p* < 0.01). We established that there was not significant effect of task and effect of age on CV of RT both “Correct Go” and “Correct Go” after “Correct NoGo” (*p* > 0.05). In all cases the interaction effect of age and task was not significant (*p* > 0.05).

### Effect of the 2 h “Go/NoGo” Task on Motivation

The performance of the 2 h “Go/NoGo” task significantly decreased the intrinsic motivation from 23.2 (5.1) to 20.2 (5.8) (*p* < 0.05) in young men, whereas intrinsic motivation remained unchanged in elderly men [24.6 (4.4) to 22.5 (4.3); *p* > 0.05]. The prolonged performance of the task did not affect the success and overall motivation in young men [16.5 (5.5) to 17.4 (6.4) for success motivation, and 3.2 (0.8) to 3.1 (0.8) for overall motivation; *p* > 0.05] and elderly men [17.2 (3.3) to 16.5 (4.1) for success motivation, and 3.1 (1.1) to 3.5 (0.5) for overall motivation; *p* > 0.05]. There were no significant differences in success, intrinsic, and overall motivation variables between young men and elderly men.

### Effect of the 2 h “Go/NoGo” Task on Mood State

There were no significant differences in mood variables before the “Go/NoGo” task between the young men and the elderly men (**Table 1**). Subjective fatigue, tension, and confusion significantly (*p* < 0.05) increased, and vigor decreased after the “Go/NoGo” task in the young men. Subjective fatigue significantly (*p* < 0.05) increased after the “Go/NoGo” task in the elderly men.

**Table 1:** Mood state before and after a 2 h “Go/NoGo” task.

	Young		Elderly	
	Before	After	Before	After
		
Parameter	Go/NoGo task		Go/NoGo task	
Anger	4.4 (0.9)	4.6 (0.7)	4.3 (0.5)	5.0 (2.5)
Confusion	4.5 (1.2)	6.7 (3.0)^∗^	4.9 (1.5)	5.9 (2.5)
Depression	4.5 (1.5)	5.4 (2.4)	4.4 (0.5)	5.5 (3.0)
Fatigue	6.3 (2.9)	12.1 (4.7)^∗^	5.4 (1.8)	7.6 (3.1)^∗^
Tension	4.8 (1.1)	5.8 (1.8)^∗^	6.3 (2.0)	5.6 (2.4)
Vigor	15.1 (3.2)	10.5 (3.5)^∗^	14.8 (2.4)	14.5 (3.3)

*Data are presented as mean (standard deviation). ^∗^p ≤ 0.05 compared with the values before the task.*

### Effect of the 2 h “Go/NoGo” Task on Cognitive Performance

In the young men, the RT and error rate of Memory Search Task (MST) significantly increased (*p* < 0.05), while the accuracy of ST increased and the accuracy of CSDT decreased in the elderly men after the 2 h “Go/NoGo” task (*p* < 0.05) (**Table 2**). The RT variable of all cognitive tasks was significantly greater in the elderly men (*p* < 0.05; *OP* > 0.90). The accuracy of ST, MT, and CSDT was greater, and the distance from the target of the Pursuit Tracking Task (PTT) was less in young men than in elderly man (*p* < 0.05) before and after the 2 h “Go/NoGo” task.

**Table 2:** Cognitive performance before and after a 2 h “Go/NoGo” task.

	Young		Elderly	
Parameter	Before	After	Before	After
		
	Go/NoGo task		Go/NoGo task	
**Simple Reaction Time Task**				
Reaction time (ms)	296.1 (34.1)	301.4 (42.9)	376.1 (82.5)#	373.4 (82.5)#
**Two-Choice Reaction Time Task**				
Reaction time (ms)	416.8 (51.6)	423.8 (51.8)	601.0 (11.3)#	637.2 (104.1)#
Accuracy (%)	95.9 (3.0)	95.9 (3.8)	97.8 (2.5)	98.8 (1.9)
**Switching Task**				
Reaction time (ms)	2059.2 (481.8)	1982.5 (286.1)	4204.8 (592.3)#	4061.8 (581.0)#
Accuracy (%)	93.4 (4.4)	93.8 (2.8)	78.7 (17.9)#	86.3 (6.2)#
**Matching Grid Task**				
Reaction time (ms)	1254.2 (287.6)	1233.3 (277.9)	2488.2 (409.8)	2643.9 (453.3)
Accuracy (%)	95.5 (5.0)	96.5 (5.3)	95.0 (6.5)	97.5 (2.7)
**Mathematical Processing Task**				
Reaction time (ms)	1789.1 (382.4)	1823.3 (435.5)	2626.9 (707.0)#	2824.8 (863.8)#
Accuracy (%)	92.5 (8.9)	94.0 (5.2)	95.0 (5.3)	96.3 (4.4)
**Manikin Task**				
Reaction time (ms)	1375.6 (454.9)	1340.8 (375.3)	3205.3 (754.4)#	3414.4 (650.5)#
Accuracy (%)	93.4 (6.2)	95.0 (1.6)	79.7 (13.5)#	84.4 (9.7)#
**Memory Search Task**				
Reaction time (ms)	749.3 (110.4)	875.6 (212.6)^∗^	1453.3 (434.4)#	1463.4 (371.4)#
Accuracy (%)	97.0 (2.3)	91.0 (7.0)^∗^	94.7 (6.7)	94.7 (3.6)
**Code Substitution Immediate Task**				
Reaction time (ms)	993.6 (213.2)	1031.7 (215.3)	2331.5 (775.8)#	2089.3 (386.4)#
Accuracy (%)	98.0 (25.1)	98.1 (1.9)	92.9 (13.0)	97.7 (2.9)
**Code Substitution Delayed Task**				
Reaction time (ms)	953.5 (190.4)	969.2 (165.7)	2154.1 (702.7)#	2432.6 (517.1)#
Accuracy (%)	94.7 (5.6)	95.3 (5.6)	80.9 (14.2)	75.0 (14.0)^∗^
**Pursuit Tracking Task**				
Distance from target (mm)	6.8 (3.2)	6.4 (1.9)	11.9 (3.9) #	11.9 (3.7)#
Time on target (%)	99.7 (0.4)	99.7 (0.6)	96.4 (4.4)	96.6 (4.8)

*Data are presented as mean (standard deviation). ^∗^p ≤ 0.05 compared with values before the task; ^#^p ≤ 0.05 compared with young men.*

### Effect of the 2 h “Go/NoGo” Task on Prefrontal Cortex Activity

**FIGURE 5:**
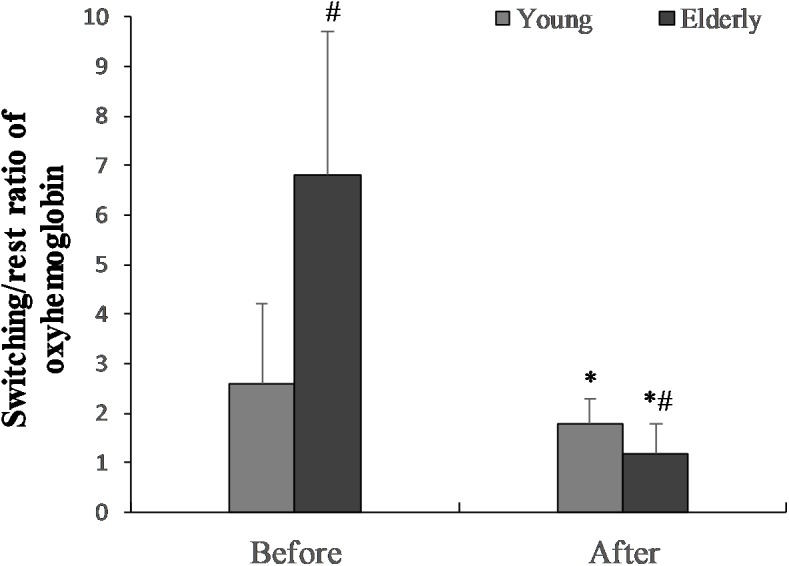
The switching/rest ratio of oxygenated hemoglobin before the “Go/NoGo” task in the young and elderly men. *^∗^p* < 0.05 compared with before the “Go/NoGo” task; *#p* < 0.05 compared with the young men.

The switching/rest ratio of oxygenated hemoglobin before “Go/NoGo” task in the elderly men was significantly greater than in the young men (*p* = 0.01) (**Figure 5**). The 2 h “Go/NoGo” task significantly decreased the switching/rest ratio of oxygenated hemoglobin for the young and the elderly men (*p* < 0.05). This ratio significantly more decreased for elderly compared to young men (*p* < 0.01). The ratio of oxygenated hemoglobin during ST after 2 h “Go/NoGo” to ST before the “Go/NoGo” task was 144.4 ± 4.2% in the young men and 33.5 ± 35.7% in the elderly men, respectively (*p* = 0.001; between the young men and the elderly men).

### Effect of the 2 h “Go/NoGo” Task on Motor Cortex Excitability

Latency increased significantly after the “Go/NoGo” task in both groups of young and elderly men (*p* < 0.05); latency was significantly greater in the elderly men before the “Go/NoGo” task (**Table 3**). The “Go/NoGo” task did not affect the MEP amplitude (*p* > 0.05) there was no difference between the MEP amplitudes of the young and the elderly men (*p* > 0.05).

**Table 3:** The effect of the 2 h “Go/NoGo” task on the motor cortex excitability.

	Amplitude (mV)		Latency (ms)	
	Before	After	Before	After
		
	Go/NoGo task		Go/NoGo task	
Young	3.1	3.8	23.5	24.4
	(1.3)	(1.4)	(1.2)	(1.0)^∗^
Elderly	2.2	2.6	24.2	24.8
	(1.3)	(1.3)	(1.4)	(1.5)^∗^

*Data are presented as mean (standard deviation). ^∗^p ≤ 0.05 compared with values before the task.*

### Effect of the 2 h “Go/NoGo” Task on Muscle Strength

Muscle strength in the young men before and after “Go/NoGo” task was respectively 51.4 (11.4) kg and 47.6 (10.6) kg (*p* = 0.004); elderly men, 44.3 (10.6) kg and 44.9 (10.3) kg (*p* = 0.031). There were significant differences before and after the 2 h “Go/NoGo” task between young men and elderly men (*p* < 0.05).

### Emotional Intelligence

There were no significant differences in emotional intelligence items of four subscales (perception of emotion, managing own emotions, managing others’ emotions and utilization of emotions) between the young men and the elderly men (*p* > 0.05) (**Table 4**).

**Table 4:** The Schutte Self-Report Emotional Intelligence Test.

	Young	Elderly
Perception of emotion	35.1 (5.9)	34.6 (2.5)
Managing own emotions	31.8 (4.5)	32.8 (3.1)
Managing others’ emotions	39.1 (4.1)	43.0 (3.4)
Utilization of emotion	14.0 (1.5)	15.8 (2.0)
All	120 (11.6)	126.4 (8.2)

### Thinking Content During the 2 h “Go/NoGo” Task

According to the DSSQ score task-related score was 22.5 ± 5.3 for young men, and 21.6 ± 4.0 for elderly men (*p* > 0.05); task-irrelevant score was 15.0 ± 5.6 for young men, and 12.0 ± 4.1 for elderly men (*p* > 0.05). Thus, there were no significant differences in thinking content during the “Go/NoGo” task between the young men and the elderly men (*p* > 0.05).

### Effect of the 2 h “Go/NoGo” Task on Perceived Workload

Physical demand increased significantly (*p* < 0.05) in the young and the elderly men during the “Go/NoGo” task; mental demand increased significantly only in the young men during “Go/NoGo” task. However, temporal demand and load effort were significantly (*p* < 0.05) greater in the young men than in the elderly men during the “Go/NoGo” task (**Table 5**).

**Table 5:** Perceived workload state during the first and the last 30 min of the 2 h “Go/NoGo” task.

	Young		Elderly	
	0–30	90–120	0–30	90–120
Mental demand	51.4 (22.3)	65.9 (29.8)^∗^	33.0 (24.3)	45.6 (20.4)
Physical demand	26.8 (20.4)	53.2 (28.8)^∗^	28.8 (14.8)	45.0 (16.7)^∗^
Temporal demand	75.9 (17.0)	77.3 (16.8)	65.0 (12.5)	56.9 (8.8)#
Load effort	68.2 (20.1)	70.0 (23.6)	56.3 (14.1)	56.9 (11.6)#
Frustration	45.0 (24.5)	47.0 (26.6)	22.6 (15.8)	33.1 (17.5)
Performance	55.0 (14.0)	55.5 (16.8)	48.8 (2.3)	52.5 (8.5)

*Data are presented as mean (standard deviation). ^∗^p ≤ 0.05 compared with first 30 min; ^#^p ≤ 0.05 compared with young men.*

### Correlation Relationships Between Variables

The average number of “Incorrect Go” errors significantly correlated with an increase in prefrontal cortical activation during ST before the “Go/NoGo” task, inversely for the young men (*r* = −0.71; *p* < 0.05), and directly for the elderly men (*r* = 0.95; *p* < 0.05); the average number of “Incorrect NoGo” errors was respectively correlated (*r* = 0.77 and *r* = 0.99; *p* < 0.05). The increase in prefrontal cortex activity after exercise during the ST, significantly correlated (*p* < 0.05) with the correct cognition task performance after the “Go/NoGo” task, directly for the young men (*r* = 0.89, *p* < 0.05) and inversely (*r* = −0.89; *p* < 0.05) for the elderly men. In addition, the internal motivation significantly correlated with the overall increase in the number of errors (*r* = −0.77; *p* < 0.05) for the young men. The percentage increase in the total number of errors for the young men correlated strongly with tension (*r* = 0.89; *p* < 0.05) and vigor (*r* = −0.77; *p* < 0.05) before exercise.

## Discussion

To our knowledge, this is the first study that has addressed the following research question: what are the differences between the young men and the elderly men in brain response (prefrontal cortex activity) and neuromuscular function (grip strength, motor control, and TMS), cognitive function (attention, executive function, memory and fast learning, and response inhibition control), and psychological variables (mood, motivation, sense of cognitive load, and thinking during exercise) during and after a PCL (2 h “Go/NoGo” task).

The first finding of our study is that during the 2 h “Go/NoGo” task, both the young men and the elderly men had a significantly similar increase in the number of “Incorrect NoGo” (inhibition) errors (the effect of age was not significant), but the number of “Incorrect Go” errors was unchanged. The RT of “Correct Go” did not significantly depend on the PCL task, but it significantly depended on age (for the elderly it was longer). Only in young RT of “Incorrect NoGo” as well as CV of RT “Incorrect NoGo” significantly increased during PCL (*p* < 0.05). Thus, contrary to our expectations, men young men showed more signs of cognitive fatigue than the elderly.

There is no doubt that during the PCL, executive function and its main elements such as concentration of attention, working memory, inhibition control, and executive flexibility were especially overloaded. This is consistent with findings by Verbruggen and Logan (2009) that response inhibition tasks require concentration of attention, working memory, and flexibility of executive function. The main target of fatigue during our PCL was the prefrontal cortex because it is the most responsible for the control of the aforementioned mechanisms (Diamond, 2013). The growing literature suggests that prefrontal contributions to executive functions cannot be analyzed in isolation from the effects of more distributed gray and white matter in healthy older adult subjects (Bettcher et al., 2016). However, it is not clear what the precise mechanisms of the origin of fatigue are because their potential contribution is considerable. For example, the neural mechanisms of mental fatigue related to cognitive task performance are more complex than previously thought and mental fatigue is not only caused by impaired activity in task-related brain regions. There is substantial evidence to support the existence of mental facilitation and inhibition systems (Ishii et al., 2014). A number of hypotheses on the mechanisms of mental fatigue origin (including self-control) have been proposed (Van der Linden et al., 2003; Ishii et al., 2014). Some of these hypotheses argue that during prolonged mental exercise, self-control resources are exhausted (Baumeister, 2014) activities of executive function and decision-making are impaired, and inhibiting processes appear in the brain (Ishii et al., 2014). Other researchers maintain that mental fatigue and self-control are highly dependent on the specifics of motivation and the reward of the task performed, especially when compared with the size of the input (Boksem et al., 2006). High internal (and perhaps external) motivation and a big reward can compensate for the manifestation of mental fatigue (Boksem et al., 2006; Braver et al., 2014) and can switch the voluntary control of task performance to be automatic (Lorist, 2008).

After a 2 h “Go/NoGo” task, handgrip strength decreased for the young men, and latency of MEPs significantly increased for both the young men and the elderly men. This clearly shows that mental fatigue caused fatigue in the neuromuscular system. However, the negative effect of mental fatigue on perception of effort reflects no greater development of central or peripheral fatigue (Pageaux et al., 2015).

Task performance strategy depends not only on the task prediction, but also on the current situation (Verbruggen and Logan, 2009). For example, if there has been an error in the case of task inhibition, then this is taken into consideration during the following trial (Verbruggen and Logan, 2009). This is consistent with our finding that after inhibition of the error, RT for the other correctly performed task was significantly longer than that under the normal conditions. Moreover, this phenomenon was exhibited more prominently by the elderly men (**Figure 3**). The second main finding of our study was that despite the motivation level at the beginning of exercise not differing between the young men and the elderly men, the young men felt more fatigue, lack of energy, and mental and temporal demand during exercise, and their intrinsic motivation was more decreased during exercise. Taken together, psychological strain in the PCL was higher for the young men than for the elderly men. This is consistent with a study by Wascher et al. (2016) that found young people experienced more fatigue from monotonous cognitive exercise than the elderly. Thus, impairment of the intrinsic motivation of young people could be a key factor in why young people felt a greater psychological fatigue because it has been clearly established that when the subjects are involved in mental activity and engage with it, they experience less mental fatigue (Shigihara et al., 2013).

When people become tired, it is more likely that they continue to perform a task using automatic control (Verbruggen and Logan, 2009). As our findings show, internal motivation decreases. We found that the smaller the internal motivation for young men, the more the number of errors increased for them in the performance of PCL. Although the young men in our study reduced their intrinsic motivation during exercise, they did not change their extrinsic motivation; we believe that they moved from a strategy of “I want” to “I have to.” This is consistent with the finding of other researchers that the “I have to” task performance strategy is more tiring than the “I want” strategy (Inzlicht et al., 2014). Moreover, this coincides with a popular principle of brain activity, namely the minimum energy (mental effort) required achieving the goal (Shenhav et al., 2013). Gergelyfi et al. (2015) concluded that mental fatigue in healthy subjects is not caused by changes in the task engagement (motivation), but is likely to be a result of a decrease in the effectiveness, or availability, of cognitive resources.

Our third main finding was that although this was unexpected, the elderly men performed some cognitive tests better (by about 10%) after PCL; they made fewer mistakes in the ST and CSIT. In sum, because many cognitive functions did not deteriorate in either the young men or the elderly men, we cannot claim that there is a significant difference between age groups. In our opinion, after “the task and motivation switch,” most cognitive tasks “recover.” This is consistent with data from other studies that manifestation of cognitive fatigue is specific, i.e., it depends not only on fatigue, but also on the specificity of tests establishing mental working capacity (Cook et al., 2014). Botvinick and Braver (2014) suggest that fatigue consists of a control mechanism that discourages individuals away from lengthy tasks and toward newer, possibly more satisfying activities. They found that after fatigue, increasing extrinsic motivation recovers to the level of performance before fatigue, and argued that this provides evidence in favor of a fatigue-induced disengagement from the task.

The fourth finding of our study was that prefrontal cortex activity increased during the ST more in the elderly men than in the young men before the “Go/NoGo” task. However, after the “Go/NoGo” task, the prefrontal cortex activity of the elderly men decreased, while in the young men it increased during the ST. The average number of “Incorrect NoGo” errors during 2 h “Go/NoGo” task significantly and directly correlated with an increase in prefrontal cortical activation during ST before the “Go/NoGo” task both in young and elderly man. However, the more the prefrontal activity increased during the ST after exercise, the greater the number of inhibition errors made by the elderly men, and the fewer the number made by the young men.

Seidler et al. (2010) showed that older adults progressively relied on cognitive brain processes for motor control (“cognitive demand”) because of structural and functional deteriorations in the motor cortical regions, cerebellum, and basal ganglia pathways. At the same time, attentional capability and other relevant cognitive resources (“cognitive supply”) are reduced because of differential degradation of the prefrontal cortex and anterior corpus callosum. This is consistent with our finding that, before the exercise, the elderly men had a more activated prefrontal cortex during a cognitive task, but after the 2 h “Go/NoGo” task, the activity decreased.

Mattay et al. (2002) found that, compared with young adults, older adults recruited additional cortical and subcortical areas for the performance of a simple RT task. Heuninckx et al. (2008) found that during isolated rhythmical hand/foot movements performed in the same direction or in opposite directions, executive, cognitive, and association brain regions were more highly activated by older adults to perform tasks that young adults performed with more automated processes. In this framework, the age-related compensatory recruitment of the prefrontal cortex, in terms of executive system, has been established (Sugiura, 2016).

Cabeza et al. (2002) established that low-performing older adults employed a similar prefrontal network as young adults, but used it uneconomically, whereas high-performing older adults responded to neural decline related to age through more recruited prefrontal cortex. However, our findings apparently contradict those of Cabeza et al. (2002), who concluded that because the prefrontal cortex is more activated before exercise than during the ST, more inhibition errors were made by the elderly men.

Greater activation of the left dorsolateral prefrontal and superior parietal cortex during working memory was more evident in middle-aged than in young adults regardless of working memory load or fatigue condition (Klaassen et al., 2014). This contradicts our findings because the activation of the prefrontal cortex of the elderly men in our study decreased after the “Go/NoGo” task.

## Conclusion

We found that young men showed greater signs of cognitive fatigue than elderly men during a PCL, young men felt more fatigue after exercise than elderly men, and elderly men performed some cognitive tests better after PCL than young men. Because of the greater mental load and (possibly) greater recruitment (mobilization) of prefrontal cortex during a 2 h “Go/NoGo” task (PCL), there was greater mental and neuromuscular performance fatigue in young compared with elderly men. Prolonged task performance decreased the switching/rest ratio of oxygenated hemoglobin for the young and the elderly men; however, greater decrease was observed for elderly than young men. Finally, baseline prefrontal cortex activity during the switching task predicted mental performance changes during demanding mental load as the more highly the prefrontal cortex was activated, the better was the inhibitory control observed in young men, and the poorer was the inhibitory control observed in elderly men.

## Ethics Statement

All procedures performed in studies involving human participants were in accordance with the ethical standards of the local Ethics Committee and with the 1964 Declaration of Helsinki and its later amendments or comparable ethical standards.

## Informed Consent

Written informed consent was obtained from all participants included in the study.

## Data Availability

The raw data supporting the conclusions of this manuscript will be made available by the authors, without undue reservation, to any qualified researcher.

## Author Contributions

AS: conception and design of the study, acquisition, analysis, and interpretation of the data, and drafting the study. AT, EM, KV, DM, DK, DV, RS, and AE: collection, analysis, and interpretation of the data. AS and SK: drafting the study and revising it critically.

## Conflict of Interest Statement

The authors declare that the research was conducted in the absence of any commercial or financial relationships that could be construed as a potential conflict of interest.
